# Tumor necrosis factor-mediated disposition of infliximab in ulcerative colitis patients

**DOI:** 10.1007/s10928-019-09652-5

**Published:** 2019-09-05

**Authors:** Sophie E. Berends, Tamara J. van Steeg, Maurice J. Ahsman, Sharat Singh, Johannan F. Brandse, Geert R. A. M. D’Haens, Ron A. A. Mathôt

**Affiliations:** 1 Hospital Pharmacy, Amsterdam UMC, Location AMC, Amsterdam, The Netherlands; 2 Gastroenterology & Hepatology Department, Amsterdam UMC, Location AMC, Amsterdam, The Netherlands; 3 Leiden Experts on Advanced Pharmacokinetics and Pharmacodynamics (LAP&P) Consultants, Leiden, The Netherlands; 4 Progenity, San Diego, CA USA; 5 Gastroenterology & Hepatology Department, Amsterdam UMC, Location VUmc, Amsterdam, The Netherlands

**Keywords:** Ulcerative colitis, Monoclonal antibody, Infliximab, Target-mediated drug disposition

## Abstract

Ulcerative Colitis (UC) is an inflammatory bowel disease typically affecting the colon. Patients with active UC have elevated tumor necrosis factor (TNF) concentrations in serum and colonic tissue. Infliximab is a monoclonal antibody directed against TNF and binds with high affinity. Target-mediated drug disposition (TMDD) is reported for monoclonal antibodies meaning that their pharmacokinetics are affected by high target affinity. Here, a TMDD model is proposed to describe the interaction between infliximab and TNF in UC patients. Data from 20 patients with moderate to severe UC was used. Patients received standard infliximab induction therapy (5 mg kg^−1^) at week 0, followed by infusions at week 2 and 6. IFX, anti-drug antibodies and TNF serum concentrations were measured at day 0 (1 h after infusion), 1, 4, 7, 11, 14, 18, 21, 28 and 42. A binding model, TMDD model, and a quasi-steady state (QSS) approximation were evaluated using nonlinear mixed effects modeling (NONMEM). A two-compartment model best described the concentration–time profiles of infliximab. Typical clearance of infliximab was 0.404 L day^−1^ and increased with the presence of anti-drug antibodies and with lower albumin concentrations. The TMDD-QSS model best described the pharmacokinetic and pharmacodynamics data. Estimate for TNF baseline (*B*_*max*_ was 19.8 pg mL^−1^ and the dissociation constant (*K*_*ss*_) was 13.6 nM. This model could eventually be used to investigate the relationship between suppression of TNF and the response to IFX therapy.

**Electronic supplementary material:**

The online version of this article (10.1007/s10928-019-09652-5) contains supplementary material, which is available to authorized users.

## Introduction

Ulcerative colitis (UC) is a chronic auto-immune disease of the colonic mucosa [1]. Patients with active UC suffer from episodes of bloody diarrhea, often accompanied with abdominal pain. Although the precise pathophysiology of UC has not been unraveled yet, tumor necrosis factor (TNF) plays an important role in mucosal inflammation.

TNF is a pro-inflammatory cytokine and has been found in increased concentrations in blood, stool, and epithelial tissue of UC patients [2–6]. In patients with inflammatory bowel disease (IBD) (i.e. UC or Crohn’s disease), serum TNF concentrations are significantly higher compared to healthy controls [3]. Also, higher TNF concentrations are present in inflamed tissue samples of IBD patients, compared to uninflamed tissue samples, suggesting a local higher inflammatory TNF load in inflamed epithelial tissue [6].

TNF is produced as a transmembrane cytokine (mTNF) and released in its soluble form (sTNF) after conversion by TNF converting enzyme (TACE) [7]. Infliximab (IFX) is an intravenously administered monoclonal antibody directed against TNF and has demonstrated to induce and maintain remission in patients with moderate to severe UC [8]. IFX can bind to both monomeric and trimeric (i.e. biologically active) sTNF with high affinity, thereby preventing binding of sTNF to TNF-receptors, receptor activation, and the subsequent inflammatory processes [9, 10].

The pharmacokinetics of IFX in IBD patients have been described extensively [11–16]. Notably, the presence of detectable anti-IFX antibodies and lower serum albumin concentrations are associated with an increased clearance of IFX. Clearance of IFX is increased up to fourfold in patients with detectable anti-IFX antibodies, often accompanied by undetectable IFX trough concentrations and clinical loss of response to IFX.

Monoclonal antibodies exhibit different pharmacokinetic properties compared to small molecules. Due to their high molecular weight and hydrophilicity, distribution to peripheral tissue is limited. Also, unlike small molecules, monoclonal antibodies are not cleared via the kidneys or liver, but via alternative pathways, primarily via proteolytic catabolism after receptor-mediated endocytosis in the reticuloendothelial system. Monoclonal antibodies are designed for a specific target, which they bind to with high affinity. Target-mediated drug disposition (TMDD) is described for monoclonal antibodies that bind with high affinity to their target, to such an extent that it affects the pharmacokinetics of the drug [17]. At low concentrations the monoclonal antibody is eliminated via lysosomal degradation through binding to its target, while at high concentrations, this elimination route becomes saturated and elimination occurs via a linear, non-saturable proteolytic pathway [18]. As a result, clearance is higher at low monoclonal antibody concentrations.

IFX binds with high affinity to its target sTNF, resulting in the formation of stable IFX-TNF complexes [9, 19].

Based on this mechanism of action of the monoclonal antibody IFX and measured sTNF concentrations, a TMDD model could be used to describe the interaction between IFX and TNF. This would provide more insight into IFX response of IBD patients with regard to not just IFX exposure, but also their TNF concentrations.

In this study, we aimed to quantify the binding of IFX to its biological target, TNF, by means of the development of a TMDD model in patients with moderate to severe UC.

## Methods

### Data and study design

Data was used from a prospective cohort study with 20 anti-TNF naive patients with moderate to severe UC, as previously reported [12]. Patients received IFX (5 mg kg^−1^) induction therapy at week 0, 2, and 6. One patient received an additional IFX administration at day 5. Serum IFX concentrations, anti-IFX antibody status, free TNF serum concentrations, C-reactive protein (CRP) and albumin concentrations were collected at day 0 (1 h after infusion), day 1, 4, 7, 11, 14 (before and 1 h after infusion), 18, 21, 28 and 42 (before infusion). Patient characteristics are summarized in Table 1. This study was approved by the local ethical committee and all patients signed informed consent before start of the study.

**Table 1:** Baseline patient characteristics

	N = 20
Sex, male (n) %	13 (65%)
Age (years), median (range)	36 (19–69)
Weight (kg), median (range)	70 (47–90)
Disease duration (years), median (range)	
Extent of ulcerative colitis, n (%):	6 (0–26)
Left-sided colitis	7 (35%)
Pancolitis	13 (65%)
Endoscopic mayo score 3, n (%)	19 (95%)
Corticosteroid refractory, n (%)	19 (95%)
Hospitalized, n (%)	7 (35%)
Concomitant thiopurines, n (%)	11 (55%)
Serum C-reactive protein (mg L^−1^), median (range)	25.3 (0.6–196.2)
Serum albumin (g L^−1^), median (range)	38 (23–45)
Faecal calprotectin (µg g^−1^), median (range)	2030 (386–13,710)
Simple clinical colitis activity index, median (range)	10 (1–15)

### Serum measurements

Serum IFX concentrations and anti-IFX antibodies were measured with a homogenous mobility shift assay (HMSA) (Prometheus Laboratories, San Diego, CA) [20, 21]. With HMSA, anti-IFX antibodies can be measured in the presence of IFX (i.e. a drug-tolerant or drug non-sensitive assay). Lowest level of quantification (LoQ) for IFX measurements was 0.06 mg L^−1^ with coefficient of variation (CV) of 12%. Free serum TNF concentrations were measured using an ultrasensitive immunoassay (Singulex, Prometheus Laboratories, San Diego, CA) and LoQ for TNF measurements was 10.0 fgs mL^−1^, with CV of 15% [22, 23].

### Pharmacokinetic/pharmacodynamic analysis

#### Model development

IFX and TNF concentrations were converted to nanomolar (nM) using their molecular weights of 149 kDa (IFX) and 52 kDa (TNF) [9, 24]. A population pharmacokinetic model for IFX has been developed previously, and was used as the starting point for this study [12]. For this model, the following covariates were evaluated: anti-IFX antibody status, albumin, CRP, and body weight. During forward inclusion, covariates were included when objective function value (OFV) decreased > 3.84 point (p = 0.05). For backwards elimination a more stringent *p* value of 0.01 was used (OFV decrease > 6.63 points). Continuous covariates were modeled according to the general equation:

1 $$P\, = \,P_{TV} \, \times \,\left( {\frac{COV}{{COV_{median} }}} \right)^{\theta }$$

where P_TV_ is the typical value of the parameter P in a patient with median covariate value (COV) and θ is the fractional change in P with each unit of deviation from the median covariate. Categorical covariates were modeled according to the general equation:

2 $$P\, = \,P_{TV} \, \times \,\theta^{COV}$$

where P_TV_ is the typical value of the parameter P and θ^cov^ is the fractional difference in P between categories.

Several structural models were evaluated to describe the data: a binding model, a full TMDD model, and a TMDD model with quasi-steady state (QSS) approximation [25]. The structural model was selected based on OFV, precision of parameter estimates, and visual inspection of goodness-of-fit plots. Inter-individual variability (IIV) was parameterized assuming exponential models. Concentrations were log-transformed and an additive error model (i.e. proportional error with log-transformed data) was used to capture residual variability.

For the binding model, serum concentrations of TNF bound to IFX were expressed in the $ERROR block as follows:

3 $$Bound\, = \,B_{max} \, \times \,\frac{{A_{C} /V_{C} }}{{K_{D} + \left( {A_{C} /V_{C} } \right)}} )$$

where *B*_*max*_ denotes the baseline serum TNF concentration, *A*_*c*_ represents the total IFX amount in serum, *V*_*c*_ the central volume of distribution of IFX and *K*_*D*_ the equilibrium dissociation rate constant.

**Fig. 1:**
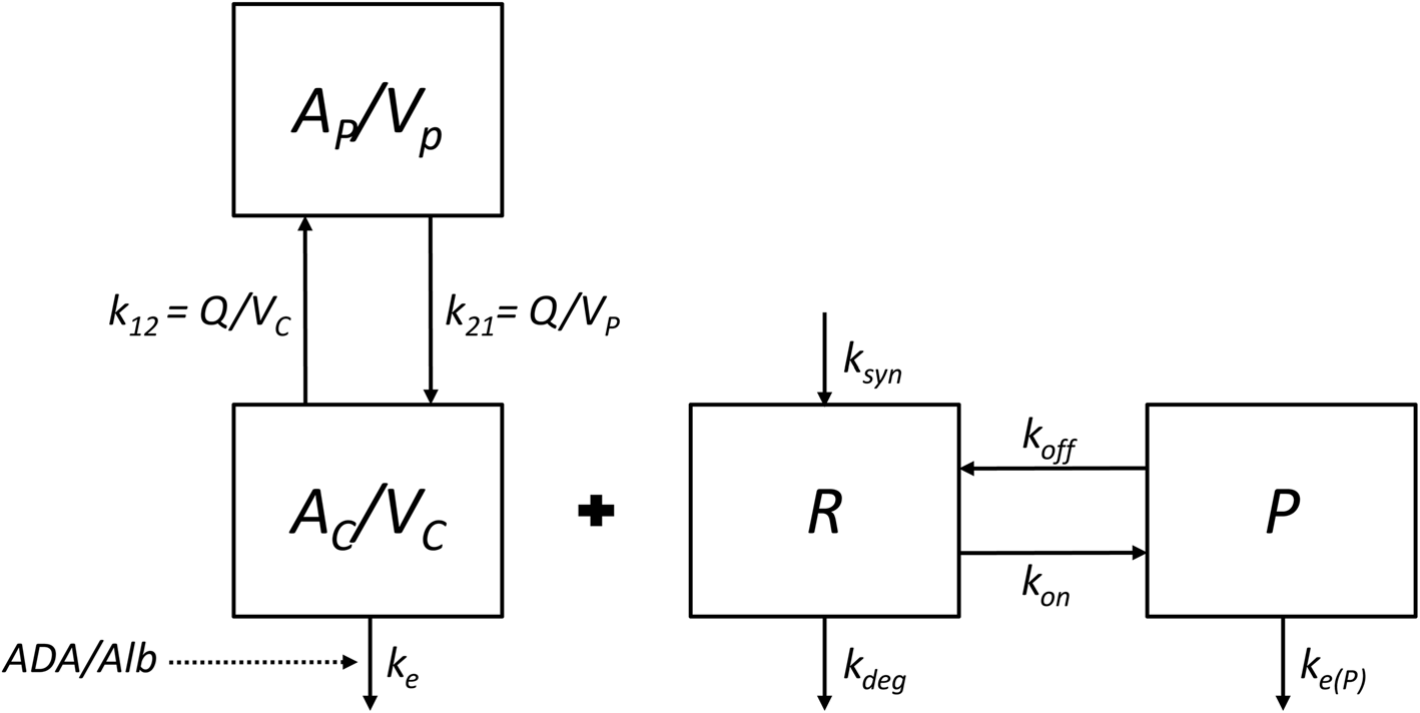
Schematic overview target-mediated drug disposition model (adapted from Mager and Jusko [26]). Symbols are defined in Table 2. *A*_*C*_ amount drug central compartment, *A*_*P*_ amount drug peripheral compartment, *k*_*12*_ first-order rate constant from the central to the peripheral compartment, *k*_*21*_ first-order rate constant from the peripheral to the central compartment, *k*_*e*_ internalization rate drug, *k*_*off*_ dissociation rate constant, *k*_*on*_ binding rate constant, *P* complex compartment, *R* receptor compartment

In the TMDD model, synthesis is represented by the zero-order rate constant *k*_*syn*_ (nM day^−1^) and degradation of TNF is represented by the first-order rate constant *k*_*deg*_ (day^−1^). TNF forms a complex with IFX with binding rate constant *k*_*on*_ (nM^−1^ day^−1^) and the complex dissociates with dissociation rate constant *k*_*off*_ (day^−1^), see Fig. 1 (adapted from Mager and Jusko [26]).

In the full TMDD model, central IFX (*A*_*c*_) and peripheral IFX (*A*_*P*_) amounts, expressed in nmol, are represented by Eqs. 4 and 5. Free TNF (*R*), and complex (*P*) concentration over time (t), expressed in nM, are represented by Eqs. 6 and 7:

4 $$\frac{{dA_{c} }}{dt}\, = \, - k_{e} A_{c} - k_{on} A_{c} R + k_{off} P\, \times \,V_{c} - k_{12} A_{c} + k_{21} A_{P}$$

5 $$\frac{{dA_{P} }}{dt} = k_{12} A_{c} - k_{21} A_{P}$$

6 $$\frac{dR}{dt} = k_{syn} - k_{on} \, \times \,\frac{{A_{c} }}{{V_{c} }}\, \times \,R - k_{off} P - k_{deg} R$$

7 $$\frac{dP}{dt} = k_{on} \, \times \,\frac{{A_{c} }}{{V_{c} }}\, \times \,R - \left( {k_{e\left( P \right)} + k_{off} } \right)P$$

where *k*_*e*_ represents the first-order elimination rate constant of IFX and *k*_*e(P)*_ represents the first-order elimination rate constant of the complex. Free IFX is distributed by first-order processes to the peripheral compartment (*k*_*12*_ and *k*_*21*_).

A QSS approximation was evaluated to improve parameter estimation in the TMDD model [25]. The main assumption of a QSS approximation is that the drug, target, and complex are in QSS, where the binding rate is balanced by the sum of dissociation (*k*_*off*_*/k*_*on*_) and internalization rates (*k*_*e(P)*_*/k*_*on*_). In contrast to a quasi-equilibrium (QE) approximation, the QSS approximation does not assume that the rate of elimination of the complex (*k*_*e(p)*_) is negligible. Instead, the dissociation constant (*K*_*SS*_) is expressed as follows:

8 $$K_{SS} = K_{D} + \frac{{k_{e\left( P \right)} }}{{k_{on} }}$$

where *K*_*D*_ denotes the equilibrium dissociation rate constant (*k*_*off*_*/k*_*on*_).

Total central IFX concentration (*C*_*tot*_), free IFX concentration (*C*), total central IFX amount (*A*_*tot*_) and the peripheral IFX amount (*A*_*P*_), are represented by Eqs. 9–12, and total TNF concentration (*R*_*tot*_), is represented by Eq. 13:

9 $$C_{tot} = A_{c} /V_{c}$$

10 $$C = \frac{1}{2}\left[ {\left( {C_{tot} - R_{tot} - K_{ss} } \right)} \right] + \sqrt {\left( {C_{tot} - R_{tot} - K_{ss} } \right)^{2} + 4K_{ss} C_{tot} }$$

11 $$\frac{{dA_{tot} }}{dt} = - \left( {k_{e} + k_{12} } \right)CV_{c} + k_{21} *A_{P} - \left( {\frac{{k_{int} R_{tot} CV_{c} }}{{K_{ss} + C}}} \right)$$

12 $$\frac{{dA_{P} }}{dt} = k_{12} CV_{c} - k_{21} A_{P}$$

13 $$\frac{{R_{tot} }}{dt} = k_{syn} - k_{deg} R_{tot} - \left( {k_{int} - k_{deg} } \right)\left( {\frac{{R_{tot} C}}{{K_{ss} + C}}} \right)$$

Due to a large difference reported *k*_*deg*_ values over multiple orders of magnitude (0.042 day^−1^ to 39.6 day^−1^) and an inability to achieve satisfactory model fit with either of these values, a sensitivity analysis was performed to assess the most likely value at which to fix *k*_*deg*_ to ensure reliable and accurate estimation of all remaining parameters [27, 28]. The sensitivity analysis was performed in NONMEM by assessing precision of parameter estimates and OFV values at each value of *k*_*deg*_. The fixed *k*_*deg*_ values ranged from 0.02 to 40.28 day^−1^, with a two-fold increase between each value and the next.

#### Model evaluation

The final model was evaluated using a visual predictive check (VPC), using 1000 simulations and the plots were stratified by compartment. Bootstrap analysis (2000 runs) was performed to test the stability and robustness of the final model parameter estimates.

#### Software

Pharmacokinetic/pharmacodynamic modeling was performed using nonlinear mixed effects modeling (NONMEM) (Icon, Dublin, Ireland, software version 7.4) with first-order conditional estimation with interaction (FOCE + I). Pearl-speaks-NONMEM (version 4.8.1, Uppsala, Sweden) and R (version 3.5.2, Vienna, Austria) were used to visualize and evaluate the model outcomes.

## Results

### Serum samples

The dataset included 214 IFX serum concentrations, and 214 TNF serum concentrations from 20 UC patients. Unbound TNF concentrations appeared inversely related to IFX serum concentrations. Directly after IFX infusion, TNF concentrations ranged from 0.27 to 2.1 pg mL^−1^ and increased to 3.5–31 pg mL^−1^ at day 42 right before the next IFX administration. Antibodies-to-infliximab were detected in 7/20 patients.

### Final model

Supported by the available data and the assumption that degradation of the IFX-TNF complex is not negligible, a TMDD model with QSS approximation best described the interaction between IFX and TNF serum concentrations. The TMDD-QSS approximation allowed the estimation of the steady state equilibrium constant (*K*_*ss*_), the complex internalization constant (*k*_*e(P)*_) and of the degradation constant TNF (*k*_*deg*_).

**Fig. 2:**
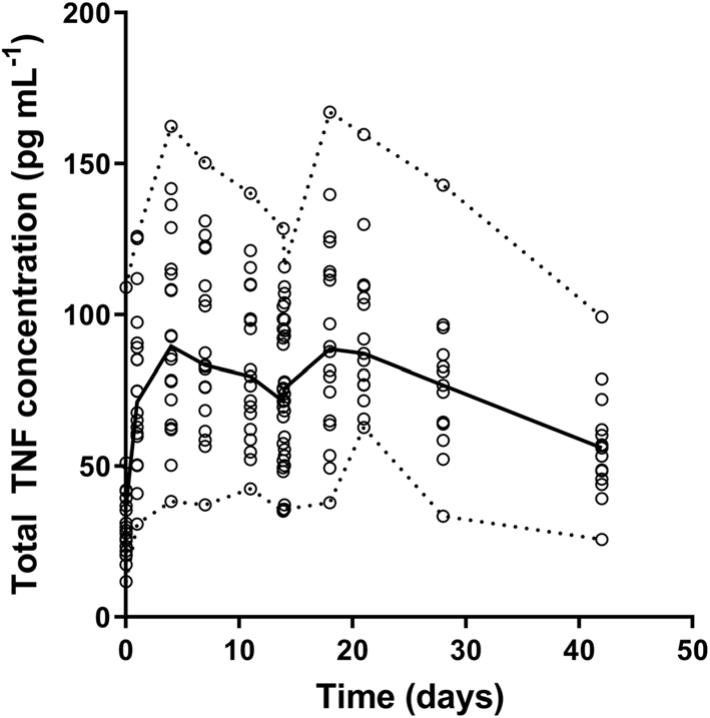
Predicted total TNF concentrations over time. Open circles represent the predicted values for total TNF concentrations. The solid line represents the median of the predicted total TNF concentrations and the dashed lines represent the lower and upper limit of the predicted total TNF concentrations

Population steady state dissociation constant (*K*_*ss*_) was 13.6 nM, *k*_*e(P)*_ was estimated to be 0.984 day^−1^ and *k*_*deg*_ was fixed to 5.12 day^−1^ based on a sensitivity analysis (see Supplementary Table 1). Median baseline TNF value (*B*_*max*_) was estimated to be 0.38 pM, which is equal to 19.8 pg mL^−1^ and comparable to literature [5]. IIV was identified for *CL*, *Vc*, *Vp*, and *B*_*max*_. Simulated total TNF concentrations are depicted in Fig. 2.

**Fig. 3:**
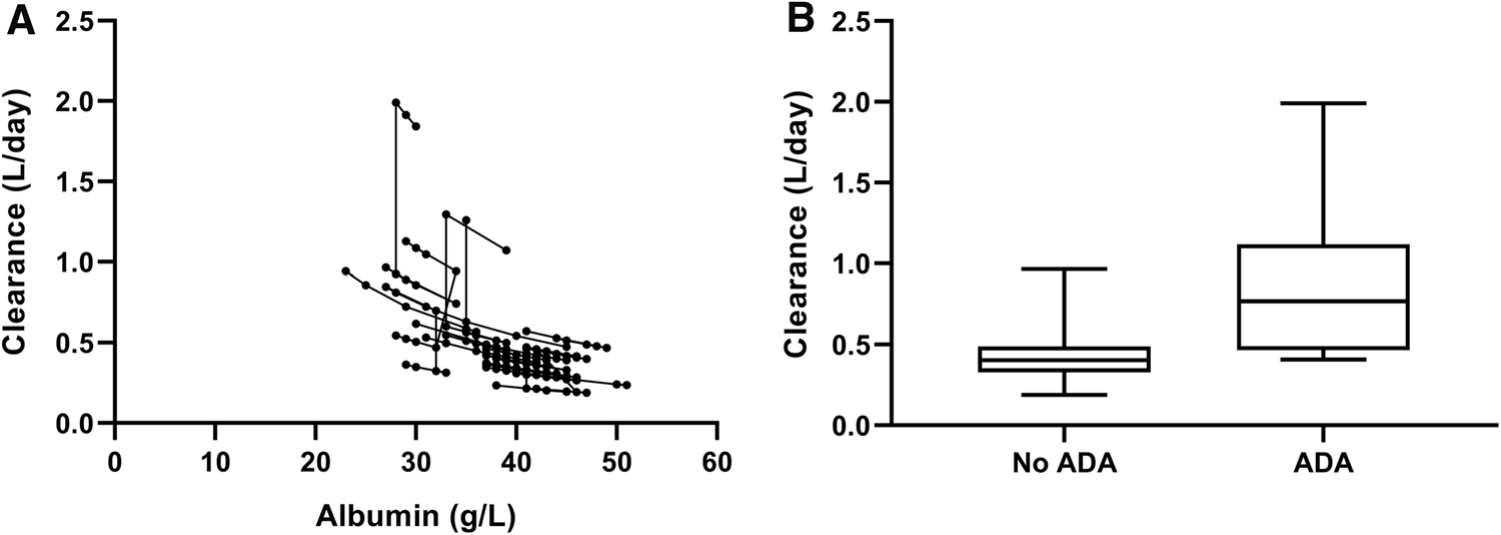
Infliximab clearance in relation to albumin concentration (**a**) and anti-drug antibodies (**b**)

Initial estimates for the typical pharmacokinetic parameters of IFX were derived from the previously developed pharmacokinetic model, and optimized during model development. The presence of antibodies-to-infliximab increased clearance of IFX by threefold (Fig. 3a). In addition, clearance of IFX ranged from 0.94 to 0.24 L day^−1^, for albumin concentrations from 23 to 51 g L^−1^ (Fig. 3b). The final parameters estimates are summarized in Table 2.

**Table 2:** Population parameter estimates of the final model and bootstrap

Parameter	Description	Estimate	RSE (%)	95% confidence interval	Shrinkage (%)	Bootstrap (95% confidence interval)
*CL* (L day^−1^)	Population clearance	0.404	9.9	0.326–0.482	–	0.406 (0.340–0.463)
*Vc* (L)	Population central volume of distribution	3.18	9.1	2.62–3.74	–	3.19 (2.85–3.52)
*Vp* (L)	Population peripheral volume of distribution	1.64	6.3	1.44–1.84	–	1.66 (1.18–2.51)
*Q* (L day^−1^)	Population intercompartmental clearance	0.344	20	0.207–0.481	–	0.336 (0.219–0.641)
ADA-CL^*^	Constant of anti-drug antibody status on clearance	2.15	12	1.64–2.67	–	2.14 (1.12–3.37)
Alb-CL^**^	Constant of median-normalized albumin level on clearance	− 1.13	36	− 1.92 to − 0.338	–	− 1.17 (− 2.10 to − 0.55)
*B*_*max*_ (pM)	Baseline TNF concentration	0.38	20	0.18–0.58	–	0.373 (0.233–0.690)
*B*_*max*_ (pg mL^−1^)	Baseline TNF concentration	19.8	–	9.57–30.2	–	19.4 (12.1–35.9)
*K*_*ss*_ (nM)	Steady-state equilibrium constant	14	24	7.09–20.1	–	13.7 (6.92–23.1)
*k*_*e(P)*_ (day^−1^)	Internalization rate complex	0.984	19	0.621–1.35	–	0.961 (0.663–1.38)
*k*_*deg*_ (day^−1^)	Degradation constant TNF receptor	5.12	–	–	–	–
*IIV*—*CL* (%)	Interindividual variability for CL	29.2	19	15.0–38.9	0.5	27.1 (12.2–39.8)
*IIV*—*Vc* (%)	Interindividual variability for Vc	22.7	16	14.1–29.1	9	21.5 (14.3–28.0)
*IIV*—*Vp* (%)	Interindividual variability for Vp	74.2	19	34.1–108	21	77.9 (41.6–153)
*Cov. CL*—*Vc* (%)	Covariance CL—Vc	12.3	106	0–21.7	–	12.1 (0–20.9)
*IIV*—*B*_*max*_ (%)	Interindividual variability for BMAX	39.2	16	22.9–51.5	3.3	37.2 (23.6–50.9)
Proportional error	Residual variability infliximab	0.210	13	0.158–0.262	–	0.199 (0.144–0.264)
Proportional error	Residual variability TNF	0.406	9	0.334–0.478	–	0.405 (0.315–0.491)

Goodness-of-fit plots for evaluation of IFX are depicted in Supplementary Fig. 1A and goodness-of-fit plots for evaluation of TNF prediction are depicted in Supplementary Fig. 1B. Population and individual predictions were randomly distributed around the line of identity for both the observed IFX and TNF concentrations. Individual weighted residuals were equally distributed along the zero line relative to individual predictions and conditional weighted residuals were equally distributed along the zero line relative to time after dose.

**Fig. 4:**
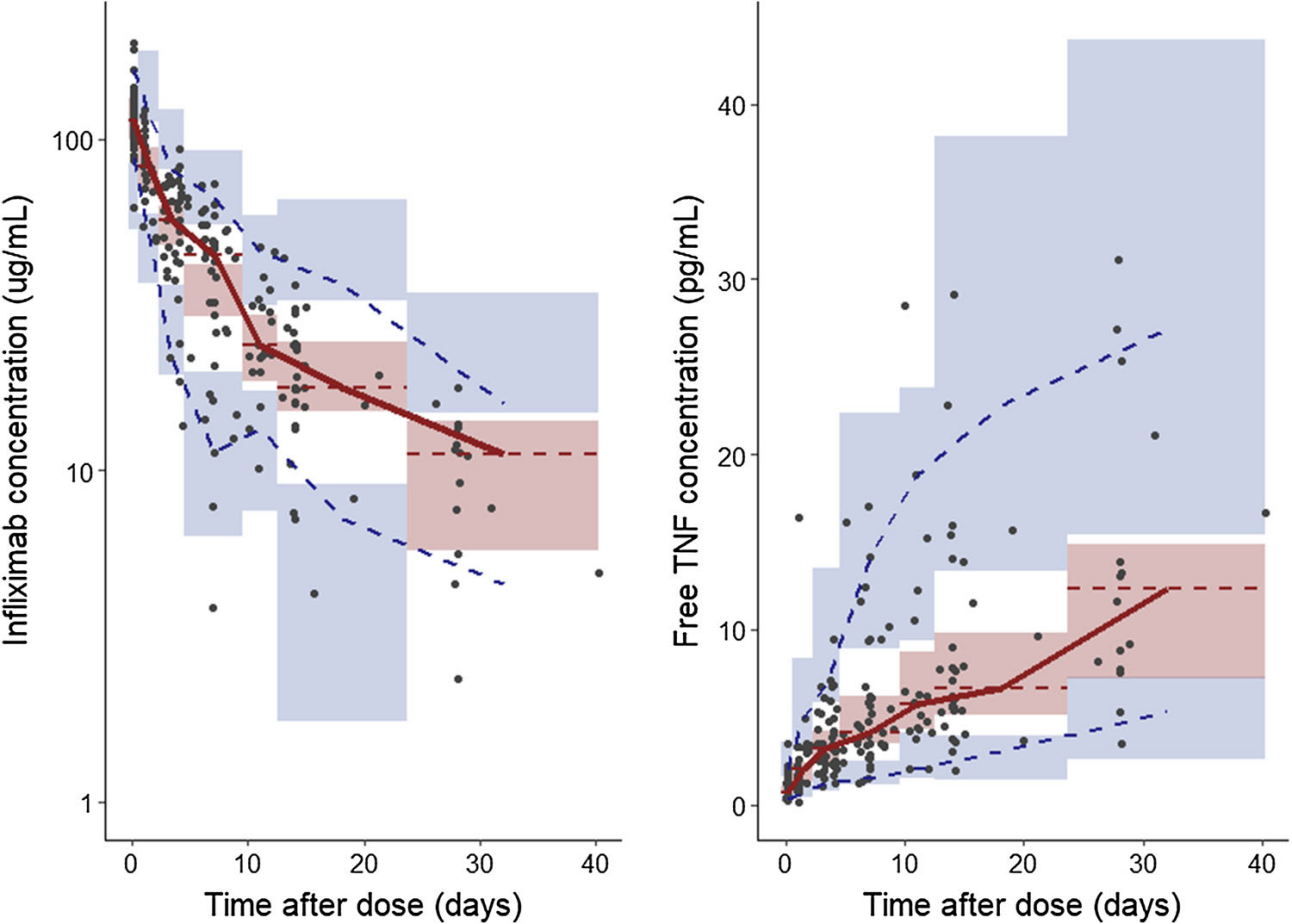
Time after dose course of model-predicted and observed infliximab and TNF concentrations. Simulations were performed (n = 1000) on the entire dataset based on the final TMDD model with QSS approximation, described by Eq. 8–13 and final parameters estimates in Table 2. Individual observations are depicted by the black dots. The solid red line represents the median of the observed data and the dashed blue lines represent the observed 5th and 95th percentiles. The red shaded area represents the 90% confidence interval of the median of the simulated data, with the red dashed lines representing the median of the simulated data per bin. The blue shaded areas represent the 90% confidence intervals of the 5th and 95th percentiles of the simulated data

Evaluation of the VPC (Fig. 4) showed that the median and 5^th^ and 95^th^ percentiles of the observed data (both infliximab and TNF data) are situated within the associated 90% confidence intervals of the prediction intervals. This indicated a good qualification of the QSS model. Bootstrap results confirmed the validity of the model (Table 1). Out of 2000 bootstrap runs for model evaluation, 1768 runs (88%) were successful and both parameters and precision were comparable to the final model.

### Simulations

Evaluation of different initial estimates showed that a lower initial estimate for *K*_*ss*_ resulted in a higher *k*_*deg*_ estimate, and higher initial estimates for *K*_*ss*_ resulted in a lower *k*_*deg*_ estimate. For that reason, different values for *K*_*ss*_ and *k*_*deg*_ were simulated and eventually, the degradation constant of TNF (*k*_*deg*_) was fixed to 5.12 day^−1^, a biologically plausible value obtained from the sensitivity analysis (Supplementary Table 1).

## Discussion

IFX is an anti-TNF agent used in patients with moderate to severe UC. In this present study we analyzed the pharmacokinetics of IFX and TNF within the first 6 weeks after start of IFX therapy in UC patients. IFX binds with high affinity to its target TNF and as a result exhibits TMDD. In this study, application of a TMDD model allowed not only the estimation of the population pharmacokinetic parameters of IFX but also the first-order elimination rate of TNF (*k*_*deg*_) and the dissociation constant of the complex of IFX and TNF (*K*_*ss*_).

A theoretical pharmacokinetic/pharmacodynamic model in which the degree of inflammation (reflected by the poor surrogate marker Crohn’s Disease Activity Index), changed by the binding of IFX to TNF in patients with CD was previously proposed [29]. More recently, the same conceptual model was evaluated for adalimumab, a subcutaneous anti-TNF agent, administered to CD patients [30]. Although both models could be applied to predict CDAI values in these patients, dependent on the complex formation between the anti-TNF agent and TNF, TNF values were not actually measured but estimated in both studies. A minimal physiology-based pharmacokinetic model with TMDD component also been has proposed to assess the interrelationship between IFX and TNF [31]. In this study, administration of a subcutaneous recombinant human-TNF infusion to boost baseline TNF, enabled the quantification of recombinant-human-TNF in the plasma of rats. A minimal physiology-based model with TMDD component was able to quantitatively describe the time-course of TNF suppression by IFX. To date, no population model has been proposed to describe the pharmacokinetics of IFX and measured sTNF in human patients.

Several structural models were evaluated to describe the pharmacokinetics of IFX and TNF in this population. First, a binding model was evaluated to describe TNF concentration–time curves, in which the binding of TNF to IFX did not influence the pharmacokinetics of the latter. However, when using a binding model to describe pharmacokinetics and dynamics, total receptor (i.e. TNF) concentrations are assumed to be constant over time. As shown in Fig. 2, total TNF concentrations increased after administration of IFX and the binding model was not able to capture this behavior adequately.

A full TMDD model was evaluated, but parameter estimates were highly dependent on initial estimates, indicating poor stability of the model and, subsequently, several approximations of the TMDD model were evaluated. Both QE and QSS approximations of the TMDD model assume an equilibrium between free drug/receptor concentrations and formation of the complex. In contrast to the QE approximation, a QSS approximation does not assume the elimination constant of the complex to be negligible. Because of the assumption that IFX-TNF complex is mainly eliminated, rather than dissociated, this approximation was deemed more applicable.

Evaluation of different initial estimates showed that a lower initial estimate for *K*_*ss*_ resulted in a higher *k*_*deg*_ estimate, and higher initial estimates for *K*_*ss*_ resulted in a lower *k*_*deg*_ estimate. To stabilize the QSS model, *k*_*deg*_ was fixed to a value which, in the absence of applicable values in literature, was based on a sensitivity analysis. Estimated *K*_*ss*_ (13.6 nM) was higher than binding affinities (*K*_*D*_) reported for IFX and TNF, which range from 0.0273 nM to 1.92 nM [19, 31, 32]. *K*_*ss*_ was expected to be higher than *K*_*D*_ values reported in literature because *K*_*ss*_ represents steady-state equilibrium by taking into account not only the equilibrium constant (*K*_*D*_ = *k*_*off*_/*k*_*on*_) but also the elimination of the complex (*k*_*e(P*)_/*k*_*on*_). Moreover, equilibrium constants are difficult to measure and are highly dependent on the type of assay performed. Furthermore, differences may exist between the in vitro and in vivo derived affinity values.

With the use of the QSS approximation, the elimination of the complex was assumed to be non-negligible and the proposed model was able to estimate the degradation constant (*k*_*e(P)*_), i.e. 0.984 day^−1^. Nonetheless, little is known about the fate of the IFX-TNF complex. It is hypothesized that the complex is recycled via the neonatal Fc-receptor (FcRn), as described for free concentrations of monoclonal antibodies. Also, the IFX-TNF complex could be subject to proteolytic degradation.

As described in Eq. 11, it was assumed that IFX is subject to nonlinear elimination. From previous literature it is however known that IFX is mainly cleared via linear elimination [11–16]. Removal of the nonlinear elimination term for IFX clearance provided a similar fit of the model to the data, suggesting that the nonlinear elimination route for IFX in the current model is indeed negligible. However, precision of parameter estimates increased indicating decreased precision. In addition, because the main purpose of this study was to describe both the interaction between IFX and TNF and the mass balance, the nonlinear elimination term was maintained in the current model.

TNF is a pro-inflammatory cytokine and after conversion released in its soluble form, i.e. sTNF. We were able to measure free sTNF concentrations in sera of patients. Although measuring free sTNF is challenging, due to the low detection level needed, Song et al. also previously reported measurements of free sTNF in patients with Crohn’s disease and found a similar trend, i.e. decrease of free sTNF concentrations after initiation of anti-TNF therapy [33].Total TNF concentrations were simulated and found increased after IFX administration (Fig. 2). This is due to the slower internalization rate of the complex (*k*_*e(P)*_) compared to the elimination rate of TNF (*k*_*deg*_), reflecting a prolonged half-life of TNF due to formation of the complex, which has been previously proposed [34]. Hence, binding of TNF to IFX protects TNF from elimination, resulting in accumulation of total TNF in the serum. This finding is in line with a recent publication that showed an increase in total TNF concentrations after start of adalimumab therapy, a subcutaneously administered anti-TNF agent [35]. In addition, it is hypothesized that the administration of IFX might trigger the conversion of membrane-bound TNF to sTNF, which results in an increased production of TNF after administration of IFX [36].

First, the study is limited by the relatively small samples size (n = 20), despite rich sampling. The main limitation of our study however is the absence of measured baseline TNF values. Baseline TNF serum concentrations have been found to be higher in responders compared to non-responders before treatment with IFX in patients with fistulizing CD [5]. In addition, TNF as predictor for treatment outcomes would be best supported by measurements of colonic tissue concentrations of TNF, as previously described by Yarur et al. [37]. Colonic tissue concentrations of TNF are significantly higher in inflamed tissue compared to matched uninflamed tissue. Also, in inflamed tissue the ratio of tissue TNF to anti-TNF are elevated compared to uninflamed tissue. With serum and colonic concentrations of both TNF and IFX, a mechanism-based model could be developed to describe the total fate of TNF in relation to IFX in this study population. Also, the potential binding of sTNF to IFX in colonic tissue could then be explored. More extensive knowledge about the total fate of TNF could then be used to investigate the relationship between suppression of TNF and the response to IFX therapy. As a result, IFX response might be predicted based on TNF concentrations and this would potentially support individualized IFX treatment.

In conclusion, we propose a TMDD model with QSS approximation to describe the interaction between IFX and TNF. This model could eventually be used to investigate the relationship between suppression of TNF and the response to IFX therapy.

## Electronic supplementary material

Below is the link to the electronic supplementary material.

Supplementary material 1 (TXT 1 kb). Supplementary file: Control stream

Supplementary material 2 (TIFF 4221 kb). Supplementary Figure 1A: Goodness-of-fit plots infliximab. Goodness-of-fit plots of the final model. The dashed lines represent the line of identity

Supplementary material 3 (TIFF 4422 kb). Supplementary Figure 1B: Goodness-of-fit plots TNF. Goodness-of-fit plots of the final model. The dashed lines represent the line of identity

Supplementary material 4 (DOCX 19 kb)
