# Supplementary material for: Tumor necrosis factor-mediated disposition of infliximab in ulcerative colitis patients
Source: J Pharmacokinet Pharmacodyn. 2019 Sep 5;46(6):543–51. doi: 10.1007/s10928-019-09652-5 (PMC6868113; doi:10.1007/s10928-019-09652-5)
Supplement: Supplementary file 4 — Supplementary material 4 (DOCX 19 kb) [file 10928_2019_9652_MOESM4_ESM.docx]

|  |  | **Run 1** | **Run 2** | **Run 3** | **Run 4** | **Run 5** | **Run 6** | **Run 7** | **Run 8** | **Run 9** | **Run 10** | **Run 11** | **Run 12** |
| --- | --- | --- | --- | --- | --- | --- | --- | --- | --- | --- | --- | --- | --- |
| OFV | Objective Function Value | -380 | -385 | -391 | -400 | -413 | -429 | -445 | -456 | -459 | -457 | -455 | -454 |
| *CL (L day^-1^)* | Population clearance | 0.408  (NA) | 0.408  (6.6%) | 0.409 (6.1%) | 0.409  (6.6%) | 0.409  (6.6%) | 0.409  (6%) | 0.407  (31%) | 0.405  (5.5%) | 0.404  (9.9%) | 0.403 (230%) | 0.403  (8.8%) | 0.403  (2400000%) |
| *Vc (L)* | Population central volume of distribution | 2.98  (NA) | 2.99  (7.2%) | 3.00 (6.3%) | 3.01  (6.4%) | 3.03  (5.9%) | 3.08  (5.8%) | 3.13  (11%) | 3.16  (5.2%) | 3.18  (9.1%) | 3.18  (1742%) | 3.17  (6.8%) | 3.16  (1100000%) |
| *Vp (L)* | Population peripheral volume of distribution | 1.90  (NA) | 1.89  (18%) | 1.87 (17%) | 1.83  (16%) | 1.78  (17%) | 1.72  (17%) | 1.66  (4.6%) | 1.63  (17%) | 1.64  (6.3%) | 1.64  (5140%) | 1.66  (17.2%) | 1.67  (503592.8%) |
| *Q (L day^-1^)* | Population intercompartmental clearance | 0.448  (NA) | 0.454  (32%) | 0.463 (36%) | 0.472  (39%) | 0.47  (40%) | 0.443  (37%) | 0.40  (13%) | 0.364  (11%) | 0.344  (20.3%) | 0.334  (2293%) | 0.337  (12%) | 0.339  (5000000%) |
| *ATI-CL^*^* | Constant of anti-drug antibody status on clearance | 2.14  (NA) | 2.14  (16%) | 2.14 (16%) | 2.14  (17%) | 2.14  (16%) | 2.15  (15%) | 2.15  (51%) | 2.15  (12%) | 2.15  (12%) | 2.15  (128%) | 2.16  (12%) | 2.16  (167592.6%) |
| *Alb-CL^**^* | Constant of median-normalized albumin level on clearance | -1.21  (NA) | -1.25  (25%) | -1.25 (21%) | -1.26  (27%) | -1.24  (21%) | -1.22  (24%) | -1.19  (81%) | -1.15  (8.8%) | -1.13  (36%) | -1.13  (1204%) | -1.11  (27%) | -1.11  (486486.5%) |
|  |  |  |  |  |  |  |  |  |  |  |  |  |  |
| *B_max_ (pM)* | Baseline TNF concentration | 0.46  (NA) | 0.32  (17%) | 0.3  (13%) | 0.3  (30%) | 0.3  (24%) | 0.3  (19%) | 0.3  (23%) | 0.4  (22%) | 0.4  (20%) | 0.4  (686%) | 0.4  (25%) | 0.4  (289351.9%) |
| *B_max_ (pg mL^-1^)* | Baseline TNF concentration |  |  |  |  |  |  |  |  |  |  |  |  |
| *K_ss_ (nM)* | Steady-state equilibrium constant | 38.6  (NA) | 53  (17%) | 52.3  (13%) | 47.9  (26%) | 41  (25%) | 34  (21%) | 26.5  (24%) | 19.4  (28%) | 13.6  (24%) | 9.04  (1582%) | 5.68  (29%) | 3.26  (585889.6%) |
| *k_e(P)_ (day^-1^)* | Internalization rate complex | 0.00463  (NA) | 0.00586  (138%) | 0.0254  (42%) | 0.0616  (35%) | 0.125  (22%) | 0.227  (18%) | 0.385  (17%) | 0.63  (17%) | 0.984  (19%) | 1.42  (513%) | 1.92  (22%) | 2.3  (111304.3%) |
| ***k_deg_ (day^-1^)*** | Degradation constant TNF receptor | **0.02 FIX** | **0.04 FIX** | **0.08 FIX** | **0.16 FIX** | **0.32 FIX** | **0.64 FIX** | **1.28 FIX** | **2.56 FIX** | **5.12 FIX** | **10.24 FIX** | **20.48 FIX** | **40.96 FIX** |
|  |  |  |  |  |  |  |  |  |  |  |  |  |  |
| *IIV – CL (%)* | Interindividual variability for CL | 28.7%  (NA) | 28.6%  (36%) | 28.6% (34%) | 28.8%  (35%) | 28.9%  (33%) | 29.1%  (33%) | 29.1%  (50%) | 29.1%  (32%) | 29.2%  (37%) | 29.2% (168%) | 29.3% (37%) | 29.3%  (1200000%) |
| *IIV – Vc (%)* | Interindividual variability for Vc | 22.6%  (NA) | 22.5%  (32%) | 22.5% (33%) | 22.5%  (33%) | 22.5%  (33%) | 22.5%  (32%) | 22.6%  (65%) | 22.7%  (31%) | 22.7%  (31%) | 22.8%  (181%) | 22.9%  (31%) | 22.9% (20939.3%) |
| *IIV – Vp (%)* | Interindividual variability for Vp | 59.6%  (NA) | 60.4%  (37%) | 60.3% (37%) | 60.3%  (37%) | 61.4%  (37%) | 64.4%  (36%) | 69%  (55%) | 73%  (38%) | 74%  (38%) | 74.8%  (797%) | 73.5%  (38%) | 72.8% (74823.5%) |
| *Cov. CL – Vc (%)* | Covariance CL - Vc | 10.9%  (NA) | 10.5%  (140%) | 10.5% (142%) | 10.6%  (34%) | 10.9%  (130%) | 11.3%  (124%) | 11.7%  (177%) | 12%  (100%) | 12.3%  (106%) | 12.3% (1427%) | 12.3%  (104%) | 12.3%  (524342.1%) |
| *IIV – B_max_ (%)* | Interindividual variability for BMAX | 38.5%  (NA) | 39.5%  (33%) | 39.6% (33%) | 39.7%  (34%) | 39.8%  (34%) | 39.8%  (34%) | 39.6%  (51%) | 39.5%  (33%) | 39.2%  (33%) | 39.1%  (315%) | 38.9%  (33%) | 38.9% (9645.4%) |
|  |  |  |  |  |  |  |  |  |  |  |  |  |  |
| *Proportional error* | Residual variability infliximab | 0.214  (NA) | 0.214  (15%) | 0.214  (15%) | 0.213  (15%) | 0.213  (15%) | 0.212  (14%) | 0.211  (13%) | 0.21  (14%) | 0.21  (13%) | 0.21  (848%) | 0.21  (12%) | 0.21  (1600000%) |
| *Proportional error* | Residual variability TNF | 0.492  (NA) | 0.486  (9.4%) | 0.478  (10%) | 0.468  (11%) | 0.453  (11%) | 0.436  (11%) | 0.42  (18%) | 0.409  (12%) | 0.406  (9.1%) | 0.408  (686% | 0.411  (12%) | 0.412  (57038.8%) |

**Supplementary table 1: Parameter estimates sensitivity analysis *k_deg_***
